# 

**Published:** 1887-02

**Authors:** 


					﻿SUBSCRIPTION PRICE, $3.00 PER YEAR, IN ADVANCE.
COLLABORATORS.
CHICAGO:
Professor E. ANDREWS, M.D., LL.D.
Professor JOHN BARTLETT, M.D.
Professor W. H. BYFORD, A.M., M.D.
Professor LESTER CURTIS, A.M., M.D.
Professor O. C. DeWOLF, A.M., M.D.
Professor E. C. DUDLEY, A.B., M.D.
Professor C. FENCER, M.D.
Professor J. N. HYDE, A.M.. M.D.
Professor E. F. INGALS, A.M., M D.
Professor W. W. JAGGARD, A.M., M.D.
Professor F. S. JOHNSON, A.M., M.D.
Professor H. a. JOHNSON, M.D., LL.D,
Professor C. W. PURDY, M.D.
Professor CHARLES G. SMITH.A.M..M.D,
Professor ELBERT WING, A.M., M.D.
MILWAUKEE, WISCONSIN:
Professor N. SENN, M.D.
WASHINGTON, D. C.:
S. O. RICHEY, M.D.
UNITED STATES ARMY:
SURGEON, D. L. HUNTINGTON, M.D
UNITED STATES NAVY:
MEDICAL DIRECTOR, J. M. BROWNE, M.D.
MEDICAL DIRECTOR, A. L. GIHON, A.M., M.D.
				

